# A vaccine targeting angiomotin induces an antibody response which alters tumor vessel permeability and hampers the growth of established tumors

**DOI:** 10.1007/s10456-012-9263-3

**Published:** 2012-03-17

**Authors:** Maddalena Arigoni, Giuseppina Barutello, Stefania Lanzardo, Dario Longo, Silvio Aime, Claudia Curcio, Manuela Iezzi, Yujuan Zheng, Irmeli Barkefors, Lars Holmgren, Federica Cavallo

**Affiliations:** 1 Molecular Biotechnology Center, University of Turin, 10126 Turin, Italy; 2 Molecular Imaging Center, Department of Chemistry IFM, University of Turin, 10126 Turin, Italy; 3 Aging Research Center, “Gabriele d’Annunzio” University Foundation, 66013 Chieti, Italy; 4 Department of Oncology and Pathology, Cancer Centre Karolinska, Karolinska Institutet, 17176 Stockholm, Sweden; 5 Molecular Biotechnology Center, Via Nizza 52, 10126 Turin, Italy

**Keywords:** Angiomotin, DNA vaccination, Vessel permeability, Antibodies, Chemotherapy

## Abstract

Angiomotin (Amot) is one of several identified angiostatin receptors expressed by the endothelia of angiogenic tissues. We have shown that a DNA vaccine targeting Amot overcome immune tolerance and induce an antibody response that hampers the progression of incipient tumors. Following our observation of increased Amot expression on tumor endothelia concomitant with the progression from pre-neoplastic lesions to full-fledged carcinoma, we evaluated the effect of anti-Amot vaccination on clinically evident tumors. Electroporation of plasmid coding for the human Amot (pAmot) significantly delayed the progression both of autochthonous tumors in cancer prone BALB-neuT and PyMT genetically engineered mice and transplantable TUBO tumor in wild-type BALB/c mice. The intensity of the inhibition directly correlated with the titer of anti-Amot antibodies induced by the vaccine. Tumor inhibition was associated with an increase of vessels diameter with the formation of lacunar spaces, increase in vessel permeability, massive tumor perivascular necrosis and an effective epitope spreading that induces an immune response against other tumor associated antigens. Greater tumor vessel permeability also markedly enhances the antitumor effect of doxorubicin. These data provide a rationale for the development of novel anticancer treatments based on anti-Amot vaccination in conjunction with chemotherapy regimens.

**Electronic supplementary material:**

The online version of this article (doi:10.1007/s10456-012-9263-3) contains supplementary material, which is available to authorized users.

## Introduction

Oncoantigens are self-molecules expressed at the tumor site that play a significant role in promoting tumor growth and can be the targets of anti-tumor vaccines [1]. Cellular [2] and DNA [3] vaccines may overcome immune tolerance and trigger a protective immune response against oncoantigens overexpressed by tumor cells [4, 5] or in tumor microenvironment [1]. Following vaccine-elicited antibody and cell-mediated immune attack, oncoantigens cannot be easily down-modulated nor negatively immunoedited as a consequence of their cancer-driving role [6]. However, while oncoantigen vaccines effectively and persistently hamper the expansion of incipient tumors, their efficacy fades away when they are administered to mice bearing advanced tumors. Their protective potential is thus restricted to tumor prevention [5, 7].

In sharp contrast to these common findings, here we show that a DNA vaccine against an oncoantigen expressed by normal, but overexpressed by endothelial cells of tumor vessels [8] also inhibits the growth of large established clinically evident tumors.

As angiogenesis drives tumor growth, inhibition of its underlying signalling pathways through vaccine-induced immune response could provide a new way of intervening with the progression of established tumors.

Induction of tumor angiogenesis is regulated by numerous pro- and anti-angiogenic factors [9]. Angiostatin specifically hampers endothelial cell migration and tumor vascularisation in mouse tumor models [10]. Its anti-migratory effects are mediated by angiomotin (Amot), one of angiostatin receptors [11]. Amot is a membrane-associated protein present on the endothelial cell surface of angiogenic tissues [8] characterized by conserved coiled-coil and carboxy termini-PDZ domains [12]. A shorter angiomotin isoform (p80) confers a hyper-migratory and invasive phenotype in transfected cells [13] and induces endothelial cell migration during angiogenesis [14]. The longer (p130) isoform localizes to tight junctions, regulates cell shape and appears to play a role in the later phase of angiogenesis [14].

We have shown that a DNA vaccine targeting Amot can overcome immune tolerance and induce a significant antibody response that mimic the effect of angiostatin. These antibodies inhibit endothelial cell migration, block tumor cell- and basic fibroblast growth factor-induced angiogenesis in the matrigel plug assay and prevent growth of transplanted tumors without impairing normal stromal or retina vessels [8]. We now show that antibodies elicited alter tumor vessel permeability and structure. These multifaceted effects of vaccine-induce anti-Amot antibodies lead to inhibition of established clinically evident mammary tumors, massive tumor perivascular necrosis, and an effective tumor antigen presentation resulting in a form of epitope spreading that induces an immune response against other oncoantigens overexpressed by tumor cells. Greater tumor vessel permeability also boosts the local accumulation of drugs and enhances their antitumor effect. These data provide a rationale for the development of fresh anticancer treatments based on anti-Amot vaccination in conjunction with chemotherapy regimens.

## Materials and methods

### Mice

Inbred female BALB/c mice, either wild type or overexpressing the transforming activated rat HER-2/neu oncogene under control of the MMTV promoter (BALB-neuT mice) [15], were bred under specific pathogen-free conditions by Biogem (Ariano Irpino, Italy) or at the Molecular Biotechnology Center (Turin, Italy). Mice overexpressing the polyoma virus middle T under control of the MMTV promoter (PyMT) were purchased from Jackson Laboratories (Bar Harbor, Maine). Mice were treated in conformity with European Guidelines and policies as approved by the University of Turin Ethical Committee.

### Culture cell lines

TUBO cells, a cloned rat Her2/neu^+^ cell line established from a lobular carcinoma of a BALB-neuT mouse [16], were cultured in Dubecco’s Modified Eagle Medium supplemented with GlutaMAX™ I, d-glucose, HEPES buffer (DMEM; Gibco, Rockville, MD), and 20% fetal bovine serum (FBS; Sigma-Aldrich, St. Louis, MO). Mouse aortic endothelial cells (MAE) transfected with human p80 Amot (MAE Amot) or empty vector (MAE vector) [11] were maintained in DMEM (Gibco) containing 10% FBS, 1% Penicillin–Streptomycin (Sigma-Aldrich) in the presence of 5 μg/ml Puromycin (Gibco). HMEC-1 (American Type Culture Collection, Manassas, VA) were cultured onto EC attachment factor (Gibco) coated tissue culture plates in EndoGRO™ medium (Millipore, Billerica, MA) supplemented with 5% FBS (Sigma) and 10 mM l-glutamine, 5 ng/ml epidermal growth factor, 1 μg/ml hydrocortisone, 0.75 U/ml heparin sulfate, 50 μg/ml ascorbic acid (all from MiIlipore) and 1% Penicillin–Streptomycin (Sigma-Aldrich).

### Tumors

Wild type BALB/c mice were challenged subcutaneously in the inguinal region with the lethal dose of 1 × 10^5^ TUBO cells. The progression of autochthonous mammary carcinomas in the mammary glands of both BALB-neuT and PyMT mice and of subcutaneous TUBO tumors was monitored weekly. Progressively growing masses with a mean diameter of >1 mm were regarded as tumors, measured with a caliper in the two perpendicular diameters, and the mean diameter was recorded. Progression of tumors was monitored until tumor masses were evident in all ten mammary glands (BALB-neuT and PyMT mice) or until a tumor exceeded a mean diameter of 10–15 mm, when mice were sacrificed for humane reasons. In a few experiments mammary glands with tumor of progressive stages and subcutaneous tumors of different sizes were collected, stored in RNA later (Sigma-Aldrich) at 4°C for 24 h, and then snap-frozen in liquid nitrogen and stored at −80°C until they were processed for morphological analysis.

### RNA extraction and quantitative real time PCR (qPCR)

Total RNA was isolated from specimens using an IKA-Ultra-Turrax^®^ T8 homogenizer (IKA^®^-Werke, Staufen, Germany) and TRIzol^®^ reagent (Invitrogen, San Diego, CA). Genomic DNA contaminations were removed from total RNA with the DNA-free kit (Ambion, Austin, TX). RNA quality was estimated by the Agilent 2100 Bioanalyzer (Agilent Technologies, Milan, Italy) and RNA was quantified with a NanoVue Plus Spectrophotometer (GE Healthcare, Milan, Italy). Total RNA was divided into aliquots and stored at −80°C till use. To detect Amot mRNA, 1 μg of DNAse-treated RNA (DNA-free™ kit) was retrotranscribed with RETROscript™ reagents (Ambion) and qRT-PCRs were carried out using gene-specific primers (QuantiTect Primer Assay; Qiagen, Chatsworth, CA), SYBR green and a 7900HT Fast Real Time PCR System (Applied Biosystems, Milan, Italy). Quantitative normalization was performed on the expression of beta-actin and the between-sample relative expression levels were calculated using the comparative delta Ct (threshold cycle number) method (2^−ΔΔCt^) with a control sample as the reference point.

### Protein preparation and immunoblotting

Total protein extracts were obtained by using a boiling buffer containing 0.125 M Tris/HCl, pH 6.8 and 2.5% sodium dodecyl sulphate (SDS). 50 μg proteins were separated by SDS-PAGE and electroblotted onto polyvinylidene fluoride membranes (BioRad, Hercules, CA). Membranes were blocked in 5% Blotto non-fat milk (Santa Cruz, CA) Tris buffered saline (TBS)-Tween buffer (137 mM NaCl, 20 mM Tris/HCl, pH 7.6, 0.1% Tween-20) for 1 h at 37°C, then incubated with appropriate primary and secondary antibodies in 1% milk TBS-Tween buffer, overnight at 4°C and for 1 h at room temperature respectively, and visualized by enhanced chemiluminescence (ECL^®^, Amersham Biosciences, Piscataway, NJ). The anti-Amot rabbit polyclonal antibody (TLE) was raised against a peptide corresponding to the 24 C-terminal amino acids of Amot, as previously described [17]; anti-Vinculin, goat anti-mouse IgG HRP-conjugated, goat anti-rabbit IgG HRP-conjugated monoclonal antibodies were all from Santa Cruz.

### In vivo treatments

Empty pcDNA3 plasmid and plasmid coding for human p80 Amot (pAmot) were generated as previously described in details [8]. Fifty μg of plasmid in 20 μl of 0.9% NaCl were injected in the quadriceps muscle of anesthetized mice. Immediately after the injection, two 25-ms trans-cutaneous electric low voltage pulses with an amplitude of 150 V and a 300-ms interval were administered at the injection site via a multiple needle electrode connected to an electroporator (Cliniporator™, IGEA s.r.l., Carpi, Italy). BALB-neuT and PyMT transgenic mice were vaccinated twice at 16th–18th and 6th–8th weeks of age, respectively. BALB/c mice injected with a lethal dose of TUBO cells were vaccinated when tumor mass reached 4 mm mean diameter and 7 days after. Few mice so treated were also injected intravenously (i.v.) once, 2 days after the second vaccination, with the maximum tolerated dose of doxorubicin (Sigma-Aldrich) (10 mg/kg of body weight).

### Antibody response

To evaluate the presence of anti-Amot and anti-Her2/neu (anti-neu) antibodies, sera from mice were collected 1 week after the last vaccination and tested by ELISA. 96 well/plates (Costar^®^, Sigma-Aldrich) were coated with 100 ng/well of recombinant human-Amot (Origene, Rockville, MD) or recombinant rat Her2/neu protein (Genway, San Diego, CA), overnight at 4°C. Coated plates were then blocked with 3% non-fat milk (Santa Cruz) in TBS-Tween buffer for 2 h at 37°C. Plates were incubate with sera diluted in blocking buffer (1:100 or 1:1,000) overnight at 4°C. To remove any non-specific antibody or excess serum protein, plates were washed 3 times with TBS-Tween buffer. HRP-conjugated anti-mouse IgG antibody (Sigma-Aldrich) was diluted 1:2,000 in blocking buffer and incubated for 1 h at 37°C. Plates were washed as described above for 6 times, followed by the addition of chromogenic 3,3′,5,5′-Tetramethylbenzidine substrate (Sigma-Aldrich). Reaction was stopped by addition of HCl 2 N and the optical density measured at 450 nm with a microplate reader (680XR, BioRad). Anti-Amot and anti-neu IgG isotype titration was performed by ELISA assay as described above using rat biotin-conjugated anti-mouse IgG1, IgG2a, IgG2b, IgG3 (BD Pharmingen, San Diego, CA) as secondary antibodies. Plates were then incubated for 30 min with streptavidin-HRP (R&D Systems, Minneapolis, MN) diluted 1:200 in TBS-Tween buffer and then reactions were carried forward as described above.

### Proliferation assay

HMEC-1 cells were plated in 96-well plates at a density of 5 × 10^3^ cells/well in EndoGRO™ medium (Millipore) with 2% FBS. After 18 h medium was removed and cell were fixed with 2.5% glutaraldehyde and stained with 0.1% crystal violet to determine t_0_. Cell proliferation was then stimulated adding fresh medium with 5% FBS. At the same time purified IgG from sera of pAmot or pcDNA3 vaccinated mice were added at the concentration of 20 μg/ml. Total IgG were purified using Melon™ Gel IgG Spin Purification kit (Thermo Scientific, Milan, Italy) according to manufacturer’s instructions. After 48 h incubation, cells were fixed with 2.5% glutaraldehyde and stained with 0.2% crystal violet. The dye was solubilized using 10% acetic acid, and optical density was measured with a Microplate Reader 680 XR (BioRad) at 570 nm wavelength. Data are presented as mean ± SEM of three replicates.

### Dynamic contrast enhanced magnetic resonance imaging (DCE–MRI)

Magnetic resonance (MR) images were acquired on anesthetized mice with an Aspect M1 MRI System (Aspect Magnet Technologies Ltd., Netanya, Israel) working at 1 Tesla. Mice were placed supine in a solenoid Tx/Tr coil with an inner diameter of 3.5 cm. A phantom filled with diluted ProHance^®^ (Bracco Imaging SpA, Milan, Italy) was included in the field of view (FOV), close to each animal, to allow correction for potential spectrometer variation. After the scout image acquisition, a T_2_-weighted (w) anatomical image was acquired with a Fast Spin Echo sequence (TR 2500 s; TE 41 ms; number of slices 8; slice thickness 1.5 mm; FOV 40 mm; matrix 128 × 128; four averages; acquisition time 2 m 40 s). DCE–MRI was performed using an axial T_1_-w, a 2D spoiled gradient echo sequence with dynamic series was acquired with three initial pre-contrast T_1_-w images and 47 dynamic post-contrast images (TR 40 ms; TE 1.8 ms; flip angle 75°; number of slices 8; slice thickness 1.5 mm; FOV 40 mm; matrix 128 × 128; one acquisition; temporal resolution 58 s per image; total examination time 50 m). B22956/1 [18], a protein-binding contrast agent (Bracco Imaging S.p.a.), is a Gd chelate with a low molecular weight linked to deoxycholic acid through a flexible spacer, and has a high affinity for serum proteins. The relaxivity of this contrast agent (CA) is 27 mM^−1^ s^−1^ at 20 MHz in human serum. It was injected at a dose of 0.05 mmol/kg in two groups of mice bearing 4 mm mean diameter TUBO tumors and vaccinated with pAmot (*n* = 6) or pcDNA3 (*n* = 3) respectively. Imaging was performed 1 day before both the first (MRI-pre) and the second (MRI-post) vaccination. Raw DCE–MRI data were analyzed by an in-house script with Matlab^®^ software (MathWorks, Massachusetts, USA). A common way of analyzing DCE–MRI images is to look at the shape of the time-intensity curve in pixels [19]. For each tumor, a region of interest (ROI) was manually drawn over the four central slices. Each voxel within the ROI was identified as enhancing if the signal intensity (SI) rose three standard deviations above the mean intensity along the first three pre-contrast images. To address the heterogeneous enhancement in tumours, the enhancing volume was divided in three sub-regions using a clustering based on k-means algorithm. An high most-enhancing, a medium- and a low-enhancing volume were thus identified for each tumor. Mean enhancement was determined by averaging the SI for these volumes. After normalization to pre-contrast SI, enhancement values were calculated at each time point of the dynamic scan, on a voxel-by-voxel basis according to the equation:

$$ {\text{SIenh}}_{{ ( {\text{t)}}}} = \frac{{\left( {{\text{SI}}_{{ ( {\text{t)}}}} - {\text{SI}}_{\text{pre}} } \right)}}{{{\text{SI}}_{\text{pre}} }} $$

wherein each voxel SI_(t)_ is the SI at a given time point, SI_pre_ is mean of the SI for the three images before the injection of the contrast agent. To delineate the shape of time–intensity curves, additional parameters were calculated voxel-by-voxel: (1) peak: the normalized peak enhancement defined as the maximal differential enhancement during the dynamic scan; (2) time-to-peak: is the time point at which the peak value was reached. Time-to-peak values were reported as the scan number, each scan being ca. 60 s long, (3) slope: is related to the enhancement increase from time zero to time-to-peak, corresponding to the rate of uptake of the CA (sec^−1^); (4) washout: is the difference in enhancement between the time-to-peak and the mean enhancement of the five last images, it is related to the amount of CA that extravasates from the tumor after the maximum enhancement (sec^−1^). Student’s *t* test was used to compare mean parameter values in each tumour before and after electroporation with *P* < 0.05 as the significant cut-off.

### Morphological observations

For the study of Amot expression in tumors, tumor cryosections (10 μm) were prepared from frozen samples using a Microm HM 560 cryostat. Sections were dried for 1 h and fixed in 4% paraformaldehyde for 10 min at room temperature and subsequently permeabilized with PBS-0.1% Triton X-100 (Sigma-Aldrich) for 5 min. Tumor sections were blocked in 5% horse serum (Sigma-Aldrich) and incubated with appropriate primary antibody for 1 h at room temperature. After 1 h incubation with secondary antibodies the sections were mounded with flouromount with DAPI (Sigma-Aldrich) and visualized using the Zeiss 700 laser scanning microscope or the Zeiss Axioplan 2 fluorescence microscope. TLE was the primary anti-Amot antibody [17]. All secondary antibodies were from Molecular Probes (Alexa Fluor, Invitrogen).

Vessel permeability was investigated by injecting 50 nm polymer microspheres (Duke Scientific, Palo Alto, CA) diluted in 0.9% NaCl (Sigma-Aldrich) to a volume of 100 μl into the tail vein 6 h before sacrifice. Tumors were then fixed in paraformaldehyde 1% for 1 h at 4°C, rinsed several times with PBS, infiltrated overnight with 30% sucrose in PBS at 4°C, embedded in Optimum Cutting Temperature (OCT; Bio Optica, Milan, Italy) compound and then frozen at −80°C. Tumor vasculature and necrosis were evaluated on tumors fixed in 10% neutralized formaldehyde solution and embedded in paraffin, or fixed in pyridoxal phosphate and embedded in OCT. 4 μm thick sections were stained with anti-CD31 antibody (Santa Cruz). Other sections were stained with hematoxylin and eosin as previously described in detail [20].

### Statistical analysis

Statistical differences were evaluated through the GraphPad software 5.0 (GraphPad Inc. San Diego, CA) by using Mantel-Cox log-rank test for the incidence of autochthonous tumors in transgenic mice; Yates’s χ^2^ test for the regression of transplantable tumors. All other statistical differences were determinate with *Student’s* *t* test.

## Results

### Amot expression increases at later stages of cancer progression

**Fig. 1:**
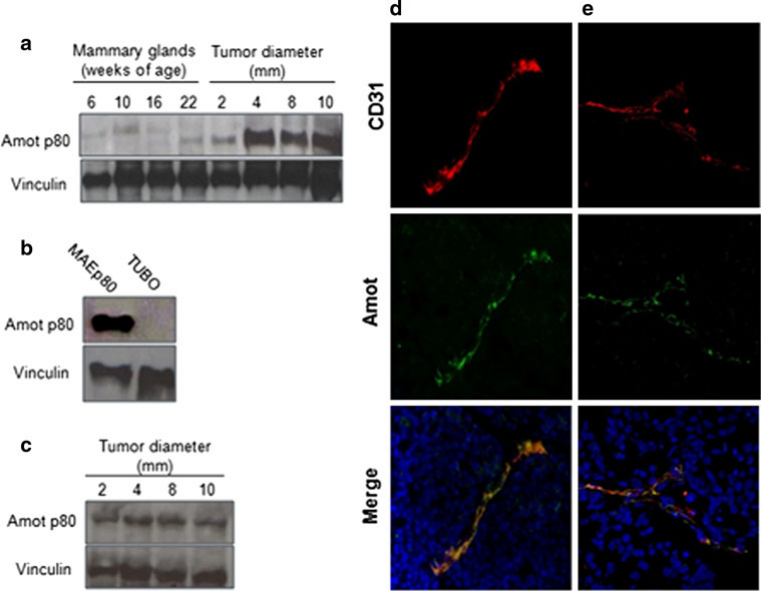
Amot expression on tumor endothelial cells and in vivo tumors. Western blot of protein extracts of: **a** mammary glands from BALB-neuT mice bearing foci of hyperplasia (week 6), in situ carcinomas (week 10) and microscopic invasive cancer (week 16, 22) and from TUBO tumors of progressive sizes (2–10 mm mean diameter); **b** MAEp80 and TUBO cells cultured in vitro; **c** TUBO tumors of progressive sizes. Immunoblots were probed with antibodies to p80 mouse Amot (*upper band*, ~80 kDa) and vinculin (*lower band*, ~100 kDa). Faint bands visible in the samples from extracts at 6, 10 and 16 weeks of age should be considered as a cross-reacting contaminant. For each determination 3 samples were analyzed. Immunofluorescence of cryosections of 5 mm mean diameter: **d** TUBO tumors growing in BALB/c mice and **e** autochthonous clinically evident mammary carcinomas from BALB-neuT mice stained with anti-CD31 (as marker of endothelial cells) and anti-Amot antibodies

Amot expression evaluated by Western blot from protein extracts of mammary glands of BALB-neuT transgenic mice bearing foci of hyperplasia (week 6), in situ carcinomas (week 10), or microscopic invasive cancer (week 22), and from autochthonous carcinomas of progressive size (from 2 to 10 mm mean diameter) (Fig. 1a), showed that the level of Amot protein increases from pre-neoplastic lesions to full-fledged lobular carcinoma (Fig. 1a). qPCR analysis on total RNA harvested from the same samples showed that Amot transcript level increases until the 22nd week (Fig. S1a), while no differences of Amot expression were found between tumors of different size (Fig. S1a). A similar pattern, albeit with a different kinetic was displayed during the progression of autochthonous carcinomas of PyMT mice (not shown). These results show that Amot transcription and expression coincides with the angiogenic switch characterized by burgeoning capillary sprouts that accompanies the progression of preneoplastic lesions towards invasive cancer [15, 21].

Amot expression levels was analyzed in in vitro cultured TUBO cells as well as in TUBO tumors grown in BALB/c mice (Fig. 1b, c). Even if Amot transcript was present (Fig. S1b), Western blot analysis showed that Amot protein was undetectable on cultured TUBO cells (Fig. 1b) while it was evident in established TUBO tumors (Fig. 1c). Immunofluorescence analysis on cryosections of established TUBO tumors (Fig. 1d) and autochthonous carcinomas of BALB-neuT (Fig. 1e) and PyMT mice (Fig. S2) disclosed Amot expression on endothelial cells of tumor vessels.

### Anti-Amot vaccination hampers the growth of autochthonous mammary carcinomas in BALB-neuT and PyMT mice

**Fig. 2:**
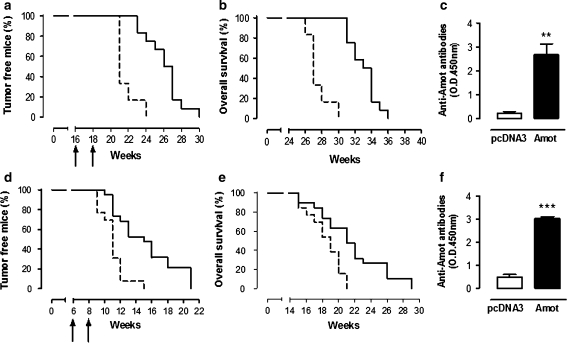
Effect of anti-Amot vaccination on mammary carcinogenesis in BALB-neuT. **a**, **b** BALB-neuT and **d**, **e** PyMT mice electroporated twice (*arrows*) with the pAmot (*black lines*, *n* = 12 BALB-neuT and *n* = 19 PyMT mice) or pcDNA3 (*dotted lines*, *n* = 11 BALB-neuT and *n* = 13 PyMT mice) plasmids. **a**, **b** *P* < 0.0001; **d** *P* = 0.0002; **e** *P* = 0.004 (*Mantel*-*Cox test*). Titers of anti-Amot antibodies in BALB-neuT (**c**) and PyMT mice (**f**) vaccinated with pAmot (*black bars*) or with pcDNA3 (*white bars*). **c** *P* = 0.008; **f** *P* < 0.0001 (*Student’s t test*)

Vaccination of BALB-neuT mice by pAmot electroporation at week 16, when the angiogenic switch accompanies the passage from in situ lesions to invasive cancer [15, 21], significantly extended tumor-free (Fig. 2a) and overall survival time (Fig. 2b). At the 25th week of age, 70% of pAmot vaccinated mice were free from palpable lesions, while all those electroporated with the empty pcDNA3 plasmid displayed at least one palpable tumor. This result is of special interest since in BALB-neuT mice anti-neu vaccination affords a major and persistent protection against incipient mammary tumors whereas it is no longer able to extend the survival time of mice if started when mice display multiple invasive microscopic carcinomas (week 16) [22]. PyMT mice constitute another model of mammary cancer. The intra-epithelial neoplasia already evident in 6-week-old mice progresses to invasive carcinoma by week 8–9 [23]. This progression is so aggressive to even minimize the potential of an effective vaccine [24]. Nevertheless, pAmot vaccination at the 6th and 8th week of age significantly extended both tumor-free (Fig. 2d) and overall survival time (Fig. 2e). When all mice electroporated with the empty pcDNA3 plasmid displayed at least one palpable tumor (week 15), 45% of those vaccinated with pAmot were still free from palpable lesions. Both BALB-neuT and PyMT mice vaccinated with pAmot showed a high level of anti-Amot antibodies in their sera (Fig. 2c, f).

### Anti-Amot vaccination hampers the growth of established transplantable tumors

**Fig. 3:**
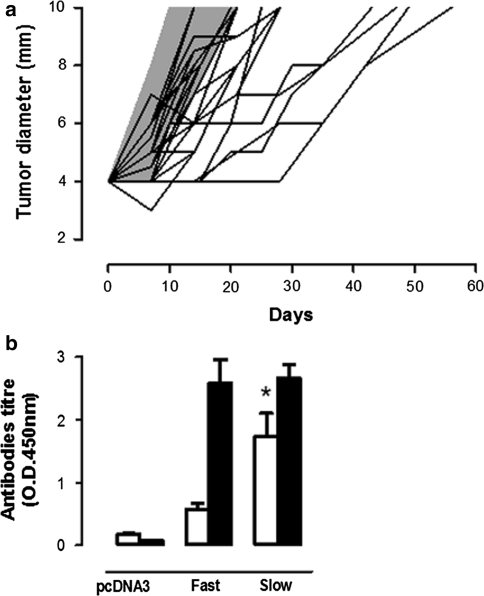
Effect of anti-Amot vaccination on the growth of transplantable TUBO tumors. BALB/c mice were challenged with TUBO cells and electroporated with pAmot or pcDNA3 both when the TUBO tumor reached 4 mm mean diameter and 7 days later. **a** Progression of TUBO tumors: each line depicts the growth of a single tumor in pAmot vaccinated mice (*n* = 29). *Grey area*: tumors growing in the controls electroporated with pcDNA3 plasmid (*n* = 28). Data were cumulated from three independent and concordant experiments. **b** Titer of antibodies against Amot (*white bars*) and against neu (*black bars*) in the sera of fast and low progressor mice electroporated with pAmot and in controls electroporated with the pcDNA3 plasmid. The titers of anti-Amot antibodies are significantly higher in slow progressor mice, *P* = 0.03 (*Student’s t test*)

To asses in greater detail the effect of pAmot electroporation on clinically evident tumors, BALB/c mice bearing a 4 mm TUBO tumor were randomly electroporated with pAmot or the empty pcDNA3 plasmid. While all the 28 mice electroporated with pcDNA3 homogeneously displayed a fast tumor progression (Fig. 3a, grey area), a scattered and slower progression pattern was found in those electroporated with pAmot. The time required by 4 mm tumors to cross a 6 mm mean diameter threshold was of 6.7 ± 0.4 days in mice electroporated with pcDNA3 compared with 13.9 ± 1.3 days for those electroporated with pAmot (*P* < 0.0001). The threshold of 10 mm mean diameter was reached in 13.9 ± 0.6 days by tumors growing in pcDNA3 electroporated mice and in 26.1 ± 1.9 days in those electroporated with pAmot (*P* < 0.0001). According to the time taken to reach this threshold, pAmot electroporated mice were divided in fast progressors, with a tumor growth pattern similar to that of control mice (*n* = 14), and slow progressors when tumors reached the 10 mm threshold after day 21 (*n* = 15). Evaluation of anti-Amot antibody titers in sera collected 7 days after the last electroporation showed that slow progressor mice are those that display the higher titer (Fig. 3b). Purified IgG from sera of pAmot vaccinated mice inhibit endothelial cell migration [8] and proliferation of human endothelial cells in vitro (Fig. S3). Moreover, in both fast and slow progressors, the anti-Amot antibodies were mainly IgG2a and IgG2b (Fig. S4), the most active subclasses in both complement- [25] and antibody-dependent cellular cytotoxicity [26].

### Anti-Amot vaccination increases tumor vessel permeability

**Fig. 4:**
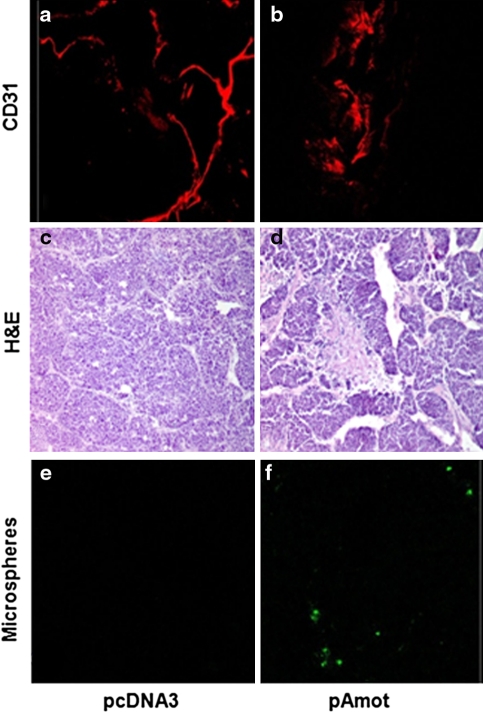
Vessel architecture, necrosis and microsphere extravasation in TUBO tumors from pAmot vaccinated mice. Architecture of the vascular system stained in red with anti-CD31 antibody in tumor sections from mice electroporated with: **a** pcDNA3 and **b** pAmot plasmids. **c** Typical areas of necrosis in a tumor from pcDNA3 electroporated mice and **d** large perivascular necrotic areas in a tumor from pAmot electroporated mice. Extravasation of fluorescent microspheres (*green spots*) extravasated in the payload of perivascular space of tumors from mice: **e** electroporated with pcDNA3 and **f** pAmot plasmids. Magnification ×200

The above findings suggest that the induction of anti-Amot antibodies directly correlates with the hampered growth of the vascularized tumors. Immunofluorescence analysis performed on tumors collected 7 days after the second pcDNA3 electroporation shows the typical irregular and convoluted structure of the branching pattern of tumor vessels (Fig. 4a). By contrast, increased mean vessel diameter and the formation of saccular and lacunar spaces are dominant features of tumor vasculature in pAmot immunized mice (Fig. 4b). A pathological evaluation of the tumors collected 7 days after the second electroporation shows that at this stage only few areas of necrosis are evident in the TUBO tumors from pcDNA3 electroporated mice (Fig. 4c). By contrast the saccular appearance of vessels in tumors from pAmot immunized mice was coupled with the presence of numerous and large areas of perivascular necrosis (Fig. 4d).

**Fig. 5:**
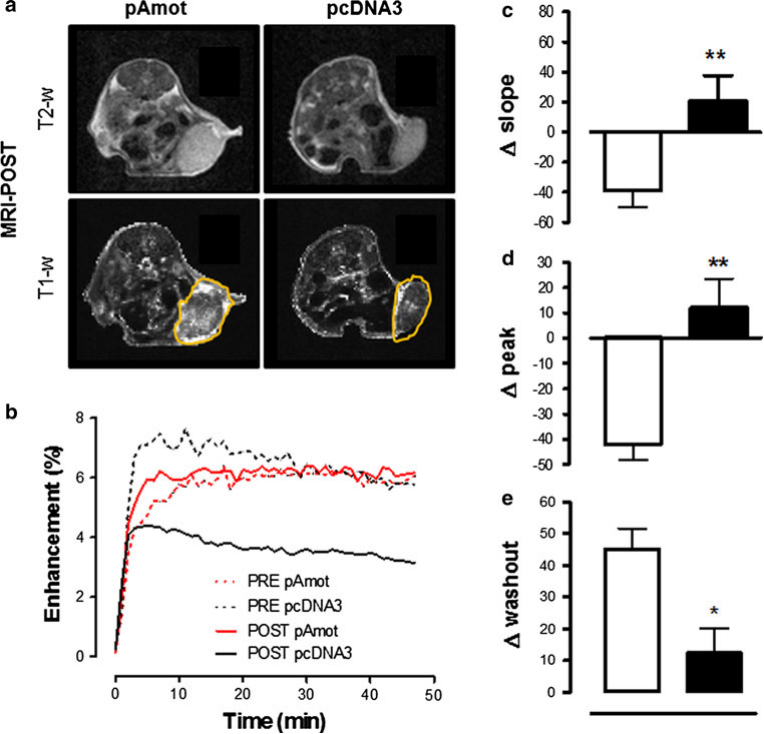
DCE–MRI of tumor vasculature on BALB/c mice bearing large vascularized TUBO tumors. **a** Representative T2-w (*upper panels*) or T1-w (*lower panels*) of MRI-POST images of mice vaccinated with pAmot (*left panels*) or pcDNA3 plasmid (*right panels*). **b** Mean curves of MRI-PRE (*dotted lines*) and MRI-POST (*continuous lines*) tumor signal intensity enhancement from mice electroporated with pAmot (*red*, *n* = 6) and pcDNA3 (*black*, *n* = 3). **c** Relative changes of slope (sec^−1^), **d** peak enhancement, and **e** washout (sec^−1^), parameters after CA administration in mice electroporated with pAmot (*black bars*) or pcDNA3 (*white bars*). ∆ slope *P* = 0.009; ∆ peak *P* = 0.007; ∆ washout *P* = 0.03 (*Student’s t*-*test*)

We next used DCE–MRI technique to assess in vivo whether the lacunar pattern acquired by tumor vessels and perivascular necrosis observed following anti-Amot vaccination were associated with a modification of morphologic and functional characteristics of tumour vasculature. DCE–MRI images obtained 48 h after the second electroporation (MRI-POST, Fig. 5a) show a higher accumulation of the CA in the tumor area of mice vaccinated with pAmot as compared to those electroporated with pcDNA3. This indication of a major alteration of vessel permeability and/or tumor perfusion following pAmot vaccination was endorsed by a semi-quantitative assessment showing that in pcDNA3 electroporated mice the amount of the CA in the tumor area is less in MRI-POST than in MRI-PRE, performed 1 day before vaccination (Fig. 5b), probably as the result of the progressive disorganization of vessels that goes along with the growth of established tumors. By contrast, in pAmot vaccinated mice the same amount of CA entered the tumor area in MRI-PRE and MRI-POST. This result points to an increased vessel permeability and fluid retention follow pAmot vaccination. In the MRI-PRE images the shape of time-intensity curves, peak enhancement, the slope and the washout parameters are similar in all mice bearing 4 mm tumors (data not shown). Despite this homogeneous starting situation, in MRI-POST the slope of the curve to reach the maximum enhancement (Fig. 5c), and the peak itself (Fig. 5d) are higher in mice electroporated with pAmot than in those electroporated with pcDNA3 plasmid. This shows that following pAmot vaccination the CA perfuses the tumor in significantly greater amount and with a faster kinetics. In addition, the low value of the washout parameter shows that retention of the CA in the tumor area is higher in pAmot vaccinated mice in comparison to pcDNA3 vaccinated ones (Fig. 5e).

Studies with fluorescent microspheres administered i.v. 7 days after the second electroporation (Fig. 4e, f) shows a significant microsphere extravasation and trapping in the payload of perivascular areas of the tumors from pAmot immunized mice (Fig. 4f). Almost no extravasation was found in pcDNA3 electroporated mice (Fig. 4e).

### Vessel alteration following anti-Amot vaccination results in a major epitope spreading

TUBO tumors overexpress the rat Her2/neu receptor [16]. Even so, their growth in BALB/c mice does not elicit an immune recognition of the tumor associated antigens nor a reactive immune response [16, 27]. However, ELISA assay showed that anti-neu antibodies were present in sera from pAmot vaccinated mice (Fig. 3b). Interestingly, both slow and fast progressors developed a significant anti-neu antibody response, indicating that pAmot vaccination was sufficient to induce tumor antigen recognition, independently from the titer of anti-Amot antibodies induced and the effect on tumor growth. Also anti-neu antibodies were mainly IgG2a and IgG2b (Fig. S3).

### Vessel permeability following anti-Amot vaccination improves chemotherapy

**Fig. 6:**
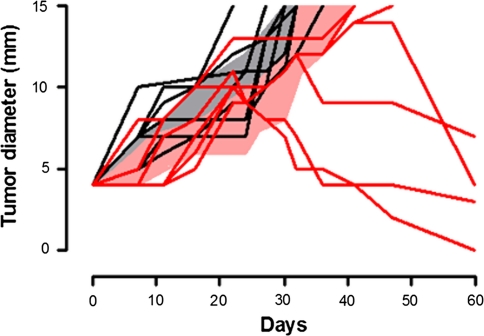
Therapeutic effect of doxorubicin in mice vaccinated against Amot. BALB/c mice challenged with TUBO cells were electroporated with pcDNA3 or pAmot when their TUBO tumor reached 4 mm mean diameter and then again 9 days later. Two days after the second electroporation, a few mice received an i.v. injection of doxorubicin. Tumor growth kinetics in mice electroporated with: pcDNA3 alone (*grey area*, *n* = 7); pcDNA3 and injected with doxorubicin (*single black lines*, *n* = 11); pAmot alone (*orange area*, *n* = 9); pAmot and injected with doxorubicin (*single red lines*, *n* = 7). A marked tumor regression was evident 4/7 (57%) of mice of the latter group. *P* ≤ 0.04 versus any other treatment group (*Yates’s χ* ^2^ *test*)

To assess whether the vessel alteration that follows anti-Amot vaccination improves the efficacy of a chemotherapy drug, BALB/c mice bearing 4 mm mean diameter TUBO tumors were electroporated twice with pAmot or pcDNA3 plasmid. Two days after the second electroporation they received a 10 mg/kg i.v. injection of doxorubicin. The combination of pcDNA3 electroporation and doxorubicin does not affect tumor growth (Fig. 6). By contrast, a significant inhibition was afforded when anti-Amot vaccination was combined with the injection off doxorubicin. Four out of seven mice showed a dramatic regression of large tumor masses, a result never observed in mice only vaccinated with pAmot.

## Discussion

The data reported here show that a DNA vaccination targeting Amot in tumor vasculature impairs the progression of established experimental mammary tumors.

This inhibition was observed in two distinct strains of cancer-prone genetically engineered mice whose females undergo mammary carcinogenesis. The aggressive progression of mammary lesions in BALB-neuT mice is driven by the activated rat HER-2/neu oncogene [28]. Atypical hyperplasia already evident in puberal BALB-neuT mice progresses into multiple foci of invasive metastatic cancer by week 16, when pAmot DNA vaccination was started [28]. Even quicker evolution of mammary lesions is driven by polyoma middle T in PyMT mice. The hyperplastic lesion evident in the mammary glands at 4 weeks of age already progresses to the stage of early carcinoma by week 8 [23], when these mice received the second pAmot vaccination. The markedly delayed onset of palpable tumors following pAmot vaccination acquires a special significance considering that anti-neu DNA vaccination is only able to elicit a marked protection when administered to BALB-neuT mice at the stage of multiple in situ carcinomas, whereas its protective ability is already marginal when it is administered at week 16 [22]. This fading of the protection is a common finding with anti-oncoantigen vaccines. The elicited response is effective until the incipient tumor is formed by a limited number of cells, while it loses its efficacy against a large number of proliferating and genetically instable tumor cells [29]. On the other hand, the growth of an established tumor relies on tumor ability to induce neovascularization and blood supply. The initial stages of a mammary tumorigenesis take advantage from the vascular tree of mammary glands, whereas as the carcinoma enlarges its dependence from newly formed Amot-positive capillary sprouts increases. Moreover, reactive lymphocytes and antibodies more readily reach their target on endothelial cells than on the cells of an established tumor [30].

Also in PyMT mice pAmot vaccination started at week 6 and 8 of age markedly extends the time of appearance of a palpable tumor and the overall survival. As comparison, in PyMT mice successful vaccination against alpha-lactoalbumin administered at the 6th week of age no more than slightly reduces the size of growing tumors [24].

pAmot vaccination performed on BALB/c mice challenged with TUBO cells and bearing a fast growing, clinically evident 4 mm mean diameter tumor was also still able to markedly impair its growth in a way directly proportional to the titer of vaccine-induced anti-Amot antibodies.

This unique ability of anti-Amot vaccination to hamper the progression of established autochthonous and transplantable tumors classifies Amot as a target oncoantigen of special interest because of its restricted spatiotemporal expression [8].

Amot overexpression was found in vessels of transplantable and autochthonous experimental mammary carcinomas [8], in vessels of human breast tumors where it correlates with the metastatic spreading [31] and in vessels associated with Kaposi’s sarcoma [11]. Systemic and local treatment with a anti-Amot monoclonal antibody prevents pathological vessel formation associated with tumor growth [32]. In BALB-neuT mice Amot expression in the mammary glands becomes prominent only following the vigorous angiogenic switch that accompanies the progression of preneoplastic lesions towards invasive cancer, characterized by burgeoning capillary sprouts [15, 21]. In the same way, Amot expression is not detectable in in vitro cultured TUBO cells. However, following a TUBO cell challenge, its expression increases at the tumor site with the expansion of tumor-induced neovascularisation. This restricted overexpression of Amot accounts for the absence of autoimmune reactions affecting the vascularisation of normal mice [8]. pAmot vaccination does not impair the fertility of immunized female mice nor their ability to deliver and feed newborn mice fully normal in number and size (not shown).

We have previously shown that the immune response that follows pAmot DNA vaccination mostly, if not completely, rests on the induction of anti-Amot antibodies [8]. This finding fits in well with the direct correlation between the titer of anti-Amot antibodies elicited by the vaccine and the intensity of the inhibition of tumor progression we have now observed. To tease apart the main features associated with the inhibition of tumor progression we exploited mice challenged with TUBO cells. First, histological analysis showed that following pAmot vaccination tumor vessels become larger and give rise to saccular and lacunar spaces. This acquired vessel pattern was associated with numerous areas of tumor perivascular necrosis. To get an assessment of how vessel modification following pAmot vaccination leads to perivascular necrosis we exploited DCE–MRI, a noninvasive technique measuring a combination of tumor perfusion and vessel permeability [33] that is currently used in clinical practice to detect early changes in the tumor induced by antiangiogenic therapies [34, 35]. We exploited the B22956/1 CA [18], a Gd-chelate with high affinity for human serum albumin in order to exploit the contrast efficiency showed by this system when combined with 1T MRI scanner [36] and the increased specificity for the detection of vascular permeability in comparison to small molecular weight Gd-complexes [37].

In pAmot vaccinated mice B22956/1 accumulates in the tumor area more than in control mice, showing that tumor vessel permeability and tumor perfusion are markedly changed. The higher DCE–MRI values of the slope of the curve to reach the peak enhancement and the higher peak itself show that B22956/1 perfuses the tumor area of pAmot vaccinated mice better and with faster kinetics. These findings suggesting that pAmot vaccination increases number and dimension of tumor vessel fenestrations are endorsed by the higher spread of fluorescent microspheres of dimensions similar to B22956/1 when bound to the human serum albumin.

The enhanced permeability attained by tumor vessels leads to a selective microsphere extravasation at the tumor site and the entrapment and persistence of the CA used in DCE–MRI in the perivascular interstitial spaces of tumor area. Histological observation shows that these features acquired by tumor vessels following pAmot vaccination correlate with multiple areas of perivascular necrosis of the tumor. Capture and processing of the dying tumor cells by antigen presenting cells recruited in the spread areas of perivascular necrosis account for the onset of a significant antibody response against neu, a major oncoantigen overexpressed by TUBO cells [7].

An almost direct correlation among the titer of anti-Amot antibodies induced by pAmot vaccination and the intensity of tumor growth inhibition was evident. However, the induction of anti-neu antibodies suggests that the inhibition of neu^+^ TUBO tumors results from a synergistic action of anti-Amot antibodies targeting tumor vasculature and anti-neu antibodies that directly inhibit TUBO cell proliferation [38, 39]. An improved antitumor effect may stem from the combined reaction against endothelia and tumor antigens [8, 40]. Nevertheless, the lack of a correlation between anti-neu antibodies and the protective effect of anti-Amot vaccination suggests a main role of anti-Amot antibodies in the protection observed. These are mainly IgG2a and IgG2b and thus, besides their direct effect on endothelial cell proliferation, they can also act by recruiting both complement and FcγRIV expressing cells [26] to damage tumor vasculature.

The increased permeability acquired by tumor vessels following pAmot vaccination also allowed an otherwise ineffective single dose of doxorubicin to induce a delay of tumor growth and a complete rejection of established TUBO tumors in 4 out of 7 mice. It is well known that antiangiogenic drugs may improve the efficacy of chemotherapy. Following the treatment with a humanized monoclonal antibody neutralizing vascular endothelial growth factor (bevacizumab) human tumors showed vessel maturation and stabilization associated with enhanced tumor perfusion, cytotoxic drug delivery and often prolonged patients survival [41]. Phase III clinical trials with bevacizumab in combination with chemotherapy have shown significant improvements in progression-free and overall survival when compared with chemotherapy alone [42].

In conclusion, present data extend to clinical evident tumors our previous observation on the ability of anti-Amot vaccination to hamper tumor onset [8]. The impaired expansion of established autochthonous and transplantable tumors, the lacunar structure and increased permeability acquired by tumor vessels, perivascular tumor necrosis and the epitope spreading leading to induction of an immune response to tumor associated antigens are multiple features triggered by the vaccination against Amot. These not only concur to impair the progression of established tumors, but also make them susceptible to an otherwise poorly effective chemotherapy. These findings prospect anti-Amot vaccination alone and in combination with conventional chemotherapy as an attractive fresh strategy for tumor therapy.

## Electronic supplementary material

Below is the link to the electronic supplementary material.

Supplementary material 1 (DOCX 14 kb)

Supplementary material 2 (TIFF 999 kb)

Supplementary material 3 (TIFF 1284 kb)

Supplementary material 4 (TIFF 275 kb)

Supplementary material 5 (TIFF 499 kb)
