# Supplementary material for: A vaccine targeting angiomotin induces an antibody response which alters tumor vessel permeability and hampers the growth of established tumors
Source: Angiogenesis. 2012 Mar 17;15(2):305–16. doi: 10.1007/s10456-012-9263-3 (PMC3338916; doi:10.1007/s10456-012-9263-3)
Supplement: Supplementary file 1 — Supplementary material 1 (DOCX 14 kb) [file 10456_2012_9263_MOESM1_ESM.docx]

**Angiogenesis**

**Supplemental Material**

**A vaccine targeting angiomotin induces an antibody response which alters tumor vessel permeability and hampers the growth of established tumors**

Maddalena Arigoni^1^, Giuseppina Barutello^1^, Stefania Lanzardo^1^, Dario Longo^2^, Silvio Aime^2^, Claudia Curcio^3^ , Manuela Iezzi^3^ , Yujuan Zheng^4^ , Irmeli Barkefors^4^, Lars Holmgren^4^ and Federica Cavallo^1^

^1^Molecular Biotechnology Center, University of Turin, I-10123 Turin, Italy;

^2^Molecular Imaging Center, Department of Chemistry IFM, University of Turin, I-10123 Turin, Italy;

^3^Aging Research Center, “Gabriele d’Annunzio” University Foundation, I-66013 Chieti, Italy;

^4^Department of Oncology and Pathology, Cancer Centre Karolinska, Karolinska Institutet, SE17176 Stockholm, Sweden.

Corresponding author:

Federica Cavallo, Molecular Biotechnology Center, Via Nizza 52, 10126 Torino, Italy. Phone: (0039) 011 670 6457; Fax: (0039) 011 236 5417; e-mail: [federica.cavallo@unito.it](mailto:federica.cavallo@unito.it)

**Supplemental figure captions**

**Fig. S1** Amot expression in tumors. Amot mRNA level was measured by qPCR from: (**a**) mammary glands of BALB-neuT mice at different weeks of age and in clinical evident tumors (2-10 mm mean diameter); (**b**) from TUBO tumors of increasing sizes (2-10 mm mean diameter). Results were calculated as fold changes (mean + SEM) relative to mammary glands with foci of atypical hyperplasia (week 6) (**a**) or to 2 mm mean diameter TUBO tumors from BALB/c mice (**b**), and normalized on β-actin. For each determination 3 samples were analyzed

**Fig. S2** Amot expression in PyMT tumors. Cryosections of tumors (5 mm mean diameter) from PyMT transgenic mice. Anti CD31 antibody as endothelial marker (upper panel), Amot staininig (middle panel) and merge (lower panel)

**Fig. S3** Anti-Amot antibodies inhibit HMEC-1 cell proliferation *in vitro*. HMEC-1 cells were incubated with purified IgG obtained from sera of mice vaccinated with pAmot (black bars) or pcDNA3 as control (white bars) for 48 hours. Cell proliferation was determined by crystal violet assay reading the absorbance of solubilized dye at 570 nm. P= 0.02 (*Student’s t test*)

**Fig. S4** Characterization of IgG subclasses of anti-Amot and anti-neu antibodies. Titer of anti-Amot (upper panel) and anti-neu (lower panel) IgG subclasses in the sera of Fast and Slow progressor mice electroporated with pAmot and in controls electroporated with the pcDNA3 plasmid
