# Supplementary figures and images for: A vaccine targeting angiomotin induces an antibody response which alters tumor vessel permeability and hampers the growth of established tumors
Source: Angiogenesis. 2012 Mar 17;15(2):305–16. doi: 10.1007/s10456-012-9263-3 (PMC3338916; doi:10.1007/s10456-012-9263-3)

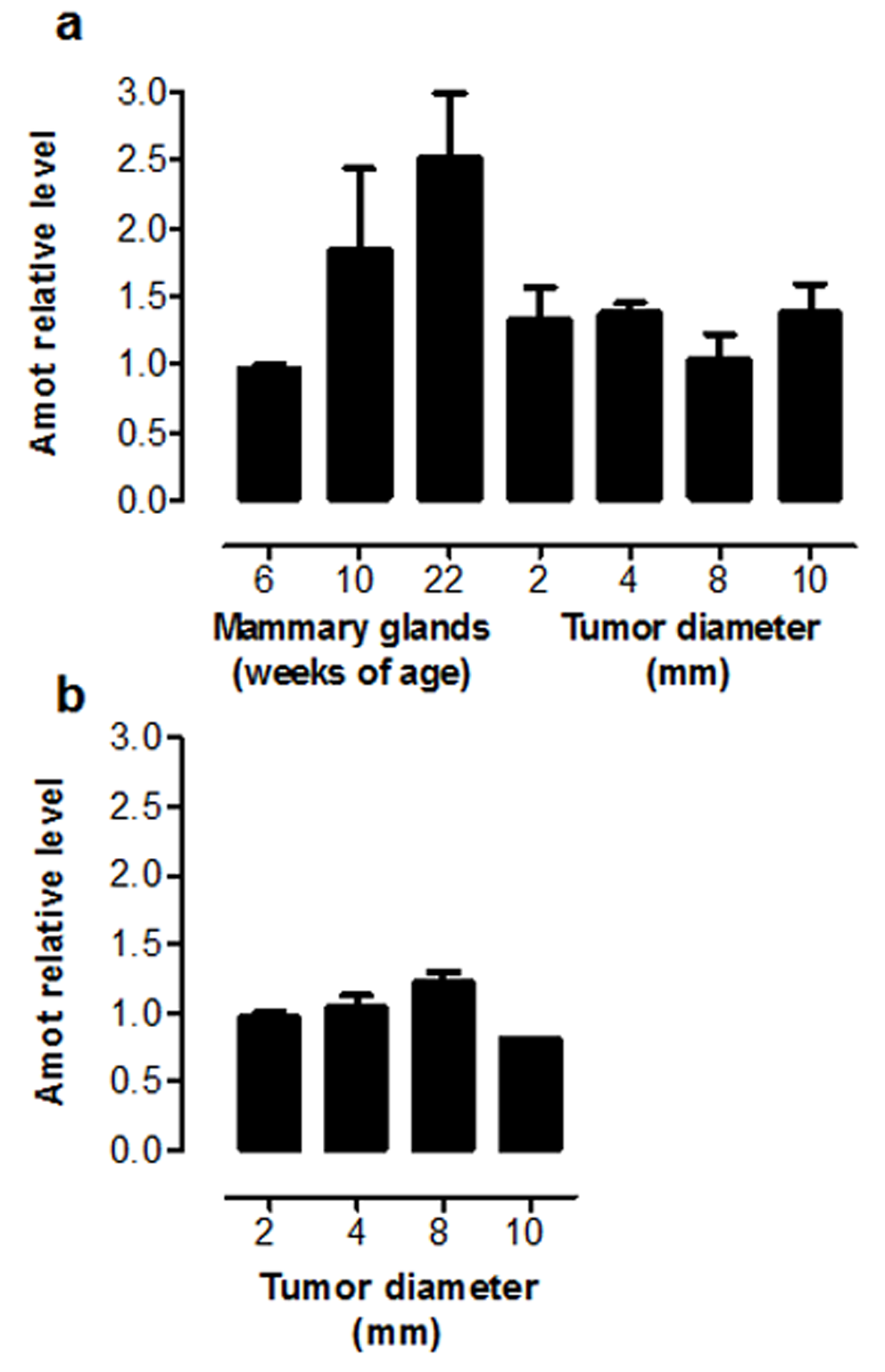

Supplement: Supplementary file 2 — Supplementary material 2 (TIFF 999 kb) [file 10456_2012_9263_MOESM2_ESM.tif]

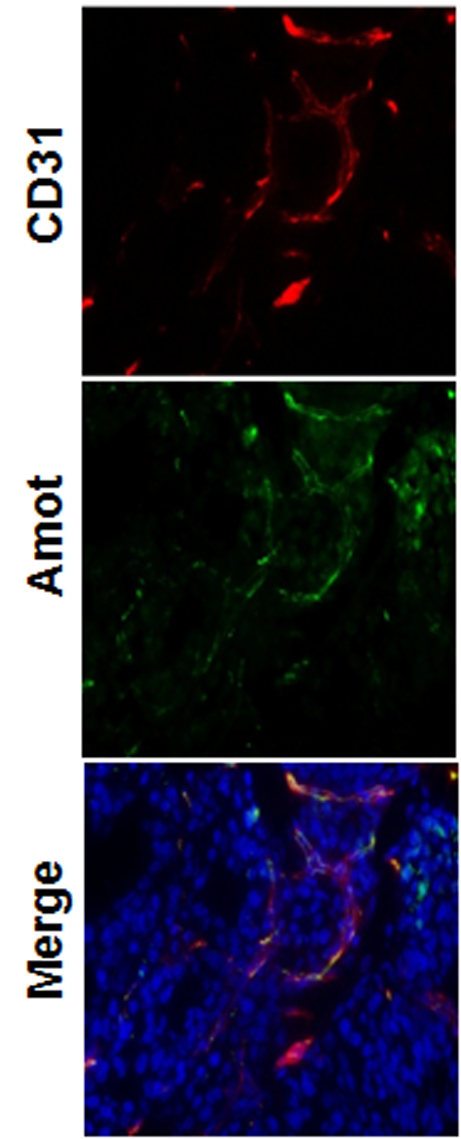

Supplement: Supplementary file 3 — Supplementary material 3 (TIFF 1284 kb) [file 10456_2012_9263_MOESM3_ESM.tif]

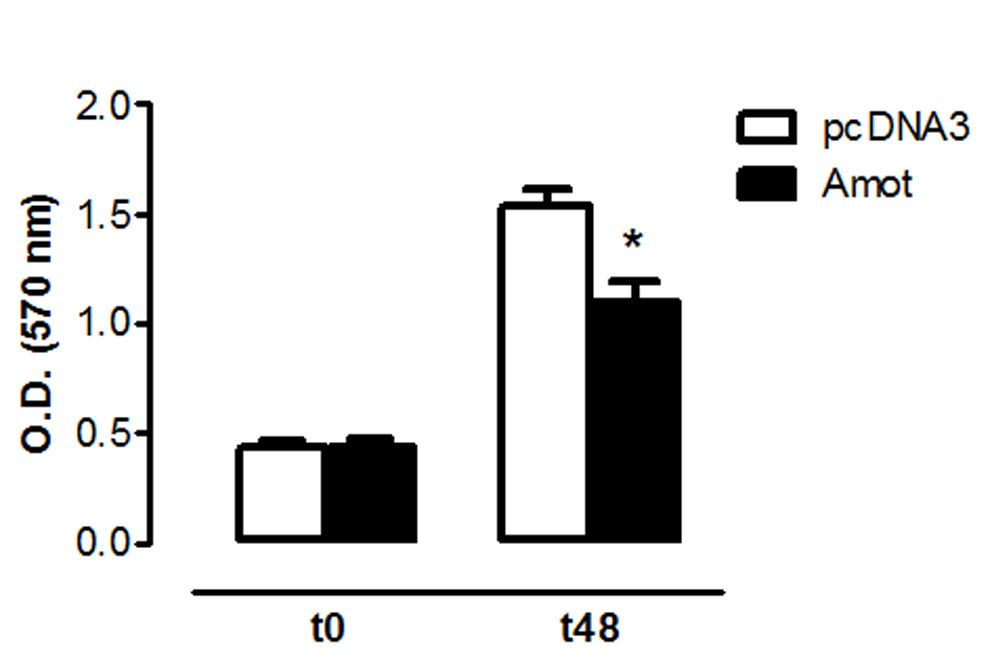

Supplement: Supplementary file 4 — Supplementary material 4 (TIFF 275 kb) [file 10456_2012_9263_MOESM4_ESM.tif]

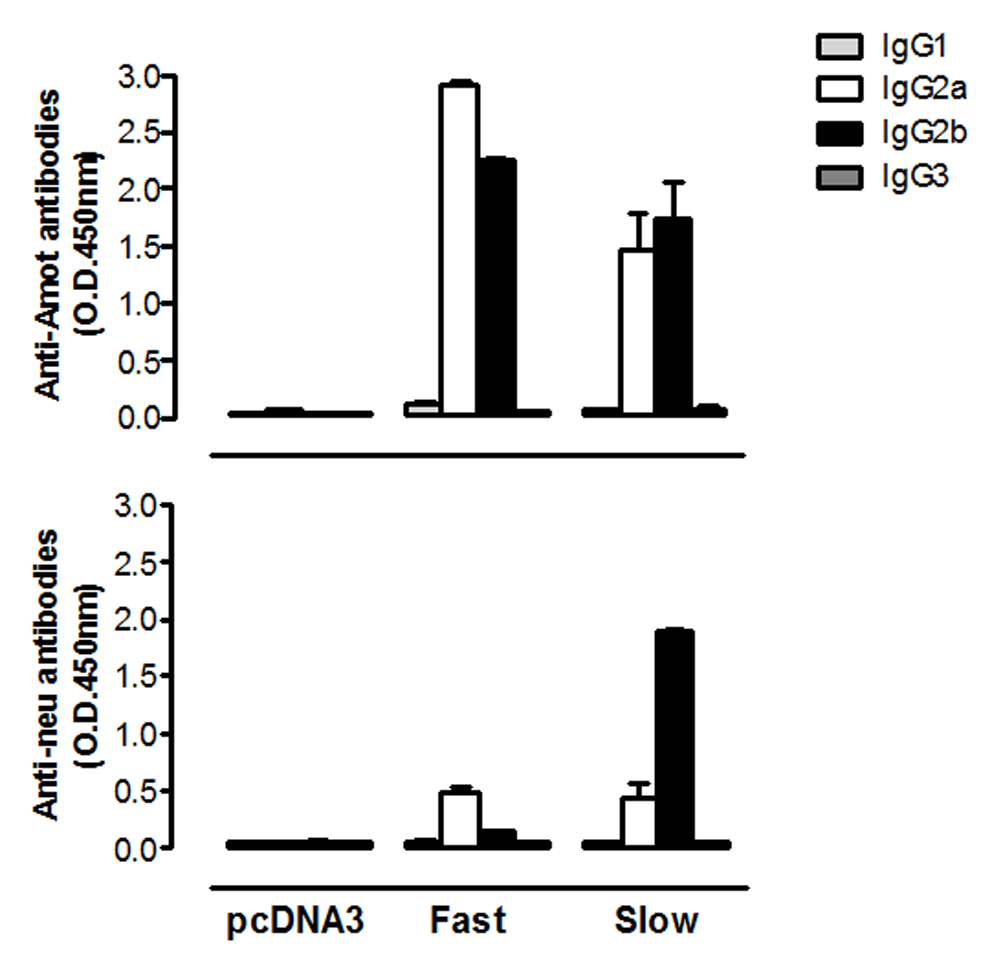

Supplement: Supplementary file 5 — Supplementary material 5 (TIFF 499 kb) [file 10456_2012_9263_MOESM5_ESM.tif]
